# Traumatic brain injury: the research continues with higher data quality evolving!

**DOI:** 10.1007/s00068-023-02255-3

**Published:** 2023-03-30

**Authors:** Marc Maegele

**Affiliations:** Cologne-Merheim Medical Center (CMMC), Department of Trauma and Orthopedic Surgery, University Witten/Herdecke, Campus Cologne-Merheim, Cologne, Germany

Traumatic brain injury (TBI) remains a significant medical and socioeconomic challenge of our times. The age-adjusted incidence across all TBI severities from population-based studies 2015–2020 ranged between 476/100,000 in South Korea to 787/100,000 in the US with temporal trends and variations still confounded by methodological diversity including inconsistent definitions, variations in data capture and interpretation [1]. To further promote and advance the uniform process for the capture and reporting of TBI epidemiology and clinical pathways, a TBI-specific data module, harmonized with other existing multi-/international instruments, was created and technically attached to the German TR-DGU database. In this focus issue of the EJTES, Younsi and colleagues report the different steps of development but also highlight the various challenges that had to be overcome along the winding road of implementation [2]. After several dry runs, the results from the pilot study are presented and put in a broader context.

The work presented by Gunning and colleagues is highly supportive of this endeavour in suggesting variability in injury severity coding to be one of the main drivers for still existing differences in reported mortality after severe TBI across international trauma centers [3]. The authors assessed 150 randomly selected patients from three international local trauma registries—50 patients from each center—and looked for inter- and intra-rater reliability of documented AIS scores. While an almost perfect result was seen for the AIS coders within the same trauma center, the opposite was true when looking at coding between the centers. In consequence, measures to improve inter-rated reliability in coding and for quality control of captured data into registries are mandatory and therefore central to the novel TBI databank within the TR-DGU.

Another key perspective of the novel TBI databank is the critical validation of current practice and the adherence to existing guidelines when managing TBI patients on a larger and more comprehensive scale. An electronic survey conducted by Lagares and the BRAINI investigators among 200 physicians from 131 hospitals in four southern European countries confirmed large variations in guideline adherence, in particular when it came to CT imaging after clinical mild TBI [4]. Another topic of ongoing discussion remains the question of whether to intubate in the field or not in TBI which is highlighted by two other contributions to this focus issue [5, 6]. Radhakrishnan and colleagues present the results from a recent review but conclusions again remain vague [5]. While the impaired level of consciousness, usually defined by GCS, is still considered as a key indicator for pre-hospital intubation and supported by most guidelines, Samuel and colleagues showed that a significant proportion of patients intubated in the field actually does not suffer from a significant injury to the brain [6]. The prevalence in their retrospective cohort with field GCS of 3, 4–8, and > 8 was 81,4%, 55,8% and 28,6%; in children below the age of 10 years with pre-hospital intubation, only half had a later proven relevant TBI. Another interesting single-center report, presented by Moyer and colleagues, evaluated the effectiveness of external ventricular drainage for increased intracranial pressure after TBI [7]. While surveys—by their nature—only provide a very limited snapshot usually missing more in-depth and relevant aspects, for example outcome, single-center experiences may not necessarily represent broader practice, may focus primarily on unique local settings and features, and therefore, cannot be generalizable. A structured and comprehensive databank would surely have the power to open up new windows of opportunity and thereby closing these gaps.

One key element of the novel TBI databank TR-DGU is outcome assessment by using the GOSE-E at 6 and 12 months with potential expansion of observation periods to later time points in the future. The focus in this respect may then clearly be directed toward the impact of rehabilitation for the overall outcome after TBI. Once broadly established and running, it is also anticipated that the novel databank will be open for the inclusion of additional laboratory datapoints and biomarkers. In the present issue, the meta-analysis by Yunlong Pei and colleagues once more confirmed the diagnostic (for traumatic intracranial lesions) and prognostic value (for mortality) of GFAP, in particular during early TBI [8].

The novel TBI databank TR-DGU, as first presented in this focus issue of the EJTES, will open up future perspectives for TBI research both on the national and international scale. Along with a series of other successful initiatives such as the pan-European CENTER-TBI project including sister projects in India and China, but also the North-American TRACK TBI initiative, it will provide not only qualitative data but also quantitative data sufficient enough to aim for future big data analysis introducing novel and innovative technologies. Large-scale international collaboration is key to overcome the still-existing gaps and hurdles in the care for the severely injured TBI patient. The first step, however, remains local motivation and active participation.

## Data Availability

No original data has been used for the present article.
